# Locked pubis symphysis in a skeletally immature patient, a case report

**DOI:** 10.1016/j.tcr.2021.100441

**Published:** 2021-02-20

**Authors:** Hany Elbardesy, Sandra O'Malley, Sinead Boran, Keith Synnott

**Affiliations:** a Department of Trauma and Orthopaedic, Cork University Hospital, Wilton, Cork, Ireland; b Department of Orthopaedic Surgery, Mater Misericordiae University Hospital, Dublin 7, Dublin, Ireland

**Keywords:** Locked pubic symphysis, Overlapped pubic symphysis, Pelvic fracture, Spine fracture, Case report

## Abstract

**Background:**

Locked symphysis pubis is an exceedingly rare pelvic injury especially in the paediatric population. This study is the first to describe this fracture in a skeletally immature patient.

**Case report:**

We report the case of a fifteen year old boy who presented to the Emergency Department (ED) after being involved in a farming injury with a left lateral compression pelvic trauma. He sustained Locked Symphysis Pubis (LSP) and internal pelvic bleeding from the right Internal Iliac Artery (IIA). He was treated successfully by selective embolization of the ILA followed by closed reduction of the LSP and percutaneous fixation of the SI joint.

**Conclusion:**

Locked symphysis pubis in the paediatric population is an exceedingly rare injury among lateral compression type pelvic fractures. Careful assessment and preoperative management planning are encouraged. Open packing of the pelvis in case of internal bleeding should be avoided in paediatric patients, only selective embolization is advocated. Closed reduction of the LSP by using the external fixator as a lever arm for reduction followed by percutaneous fixation of the SI joint. Moreover, changing the patient position to prone position followed by posterior lumbar spine stabilisation is our preferred method of treatment.

## Introduction

Locked pubic symphysis (LPS) occurs due to lateral compression injury. It is defined by overlapping of one pubic bone over the contralateral pubis [1,2]. The incidence of LPS is extremely rare among lateral compression type pelvic fractures [3]. Egger was the first to describe this type of injury in 1952 [4]. To date, there are twenty-one cases in the literature since the original description [1,2,[5], [6], [7]]. These injuries often stem from high energy blunt trauma, usually in consequence of motor vehicle accidents [8]. The mechanism of injury is hyperextension and adduction or abduction of the femur that produces a lateral compression force to the pelvis. By further compression of the pelvis, the tension on the iliofemoral ligament locks the femoral head in the acetabulum. This force may be transmitted to the pubic symphysis and cut the pubic symphysis ligaments. In theory, internal or external rotation of the femur displaces the pubic bone posterior or anterior to the contralateral unbroken pubic bone [4,5,9,10]. The morbidity and mortality are significantly high from the complications of pelvic ring fractures and other associated injuries [2,11]. Some authors advocate an initial attempt with closed reduction [12], otherwise, the guidance for treatment of LPS is limited.

One technique uses the femur as a lever by locking it in flexion, abduction, and external rotation. The iliofemoral ligaments are thought to hold the femoral head within the acetabulum and to allow reduction with a gentle abduction and rocking motion of the affected extremity [4]. Other authors have mentioned the risk of femoral fracture associated with this reduction technique hence, they advocated external rotation of the hemipelvis with force applied mostly to the iliac crest [12]. A second closed reduction technique is to apply lateral compression to the pelvis while instantaneously applying a posteriorly directed force to the symphysis [13,14]. If closed treatment is unsuccessful, or if it is not possible because of other injuries [5], open reduction should be attempted. Open reduction has been reported with [5], or without [15,16], internal fixation. We describe a polytrauma case with LPS in a skeletally immature child. There is only one other case report describing a similar injury pattern [17]. No other cases in literature documented the LSP in children. This case is reported according to SCARE criteria [18].

## Case report

A fifteen year old male presented to the Emergency Department (ED) at Cork University Hospital (CUH), a level one trauma centre serving the southeast of Ireland, after being crushed under the front wheel of a tractor on a farm. There was no significant past medical or surgical history.

Initial management followed standard Advanced Trauma Life Support (ATLS) protocol [19], and Orthopaedic Association Audit standards for Trauma (BOAST) guidelines for pelvic fracture [20].

**Fig. 1:**
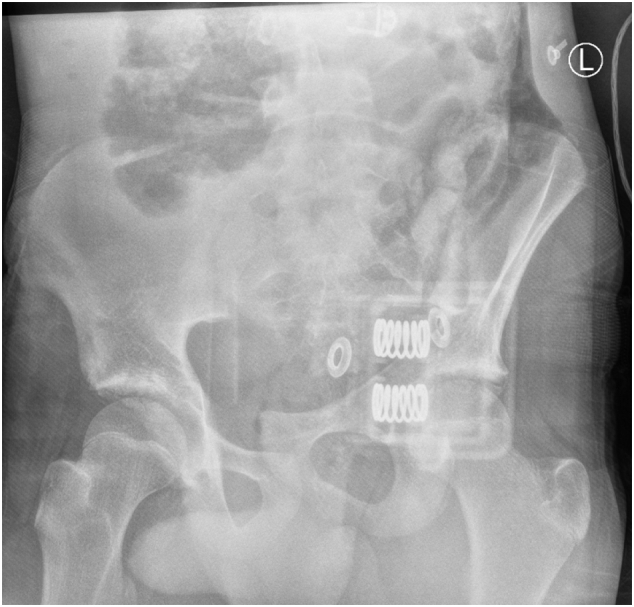
Plain films demonstrated fractures left sacrum and iliac bone and LSP.

**Fig. 2:**
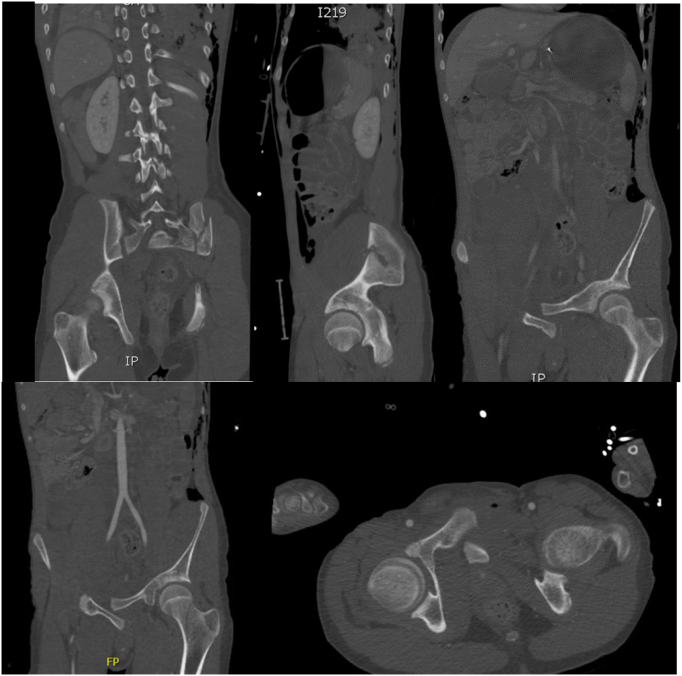
CT scan of the pelvis shows displaced fracture of the left iliac bone, commuted left sacral ala fracture, minimally displaced right-sided sacral ala fracture and complete rupture of the pubic symphysis with LSP.

**Fig. 3:**
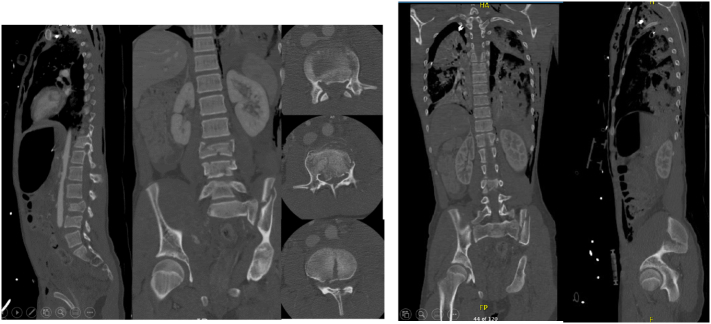
CT scan of the lumbosacral spine shows complete burst fracture of the L3 vertebral body extending into both pedicles, with significant 1.2 cm osseous retropulsion within the canal and resultant compression of the cauda equina nerve roots, further burst fracture of the L 4 vertebral body and minimally displaced transverse process fractures of the right L1-L4 vertebrae.

**Fig. 4:**
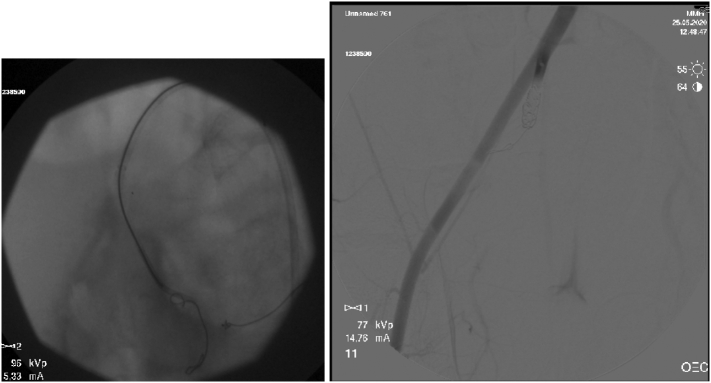
Selective embolisation of the right internal iliac artery by using 6 mm pushable Azur CX coils.

On admission Glasgow Coma Scale (GCS) was 10, the patient was intubated by the Emergency team after deterioration of his concousness level. We applied a chest tube after we had done needle decompression for left side tension pneumothorax. He underwent emergency embolization of the internal iliac artery. He responded well and the haemodynamic stability was achieved. On examination the left leg was noted to be internally rotated. Dorsalis pedis pulses were preserved bilaterally. Plain films demonstrated fractures of the left sacrum and iliac bone, a locked symphysis pubis (Fig. 1) and fracture of the distal left femur. Computed tomography (CT) scan of the pelvis (Fig. 2) demonstrated multiple fractures; displaced fracture of the left iliac bone, commuted left sacral ala fracture, minimally displaced right-sided sacral ala fracture with plastic deformation of the posterior crescent fracture configuration which was not fully extended through the iliac wing, and complete rupture of the pubic symphysis with LSP. CT scan of the lumbosacral spine (Fig. 3), showed a complete burst fracture of the L3 and L4 vertebral bodies and minimally displaced transverse process fractures of the right L1-L4 vertebrae. The surgical treatment for this young patient was a challenge. The patient was haemodynamically unstable on admission. We arranged a selective coil embolisation of the internal iliac artery by using multiple 6 mm pushable Azur CX coils (Fig. 4) to stop the bleeding after failure of all measurements of resuscitation.

A multidisciplinary team (MDT) including National Spine Injuries Unit and pelvic trauma specialist were consulted and the patient was transferred to the National Spine Injuries Unit, Mater Misericordae Hospital Dublin for definitive management.

The decision for a closed reduction to unlock the LSP by using the external fixator (Hoffman 2) as a lever arm for reduction was made as the best option for a skeletally immature haemodynamically unstable patient. The Illiosacral Joint (ISJ) was fixated with two illiosacral cannulated screws.

### Our technique

**Fig. 5:**
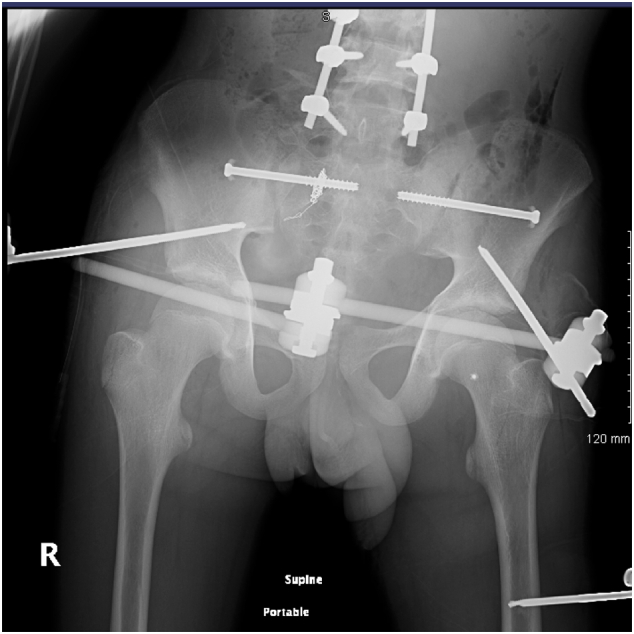
post operative radiograph with external fixator and two sacoilliac screws in situ and clips for the posterior branch of the right internal iliac artery.

**Fig. 6:**
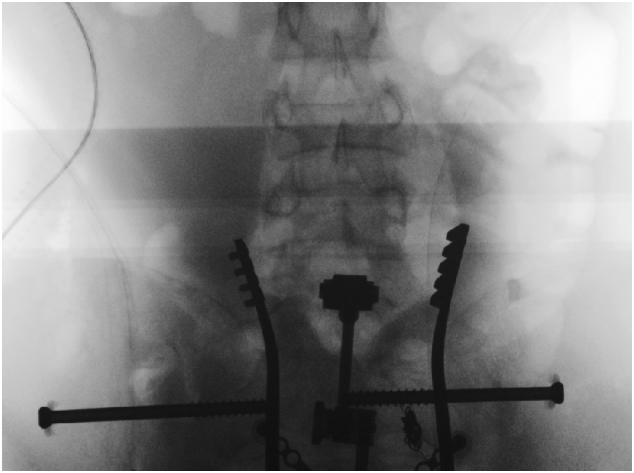
intraoperative radiograph shows fixation of the sacroilliac joint with two percutanous cannulated screws.

**Fig. 7:**
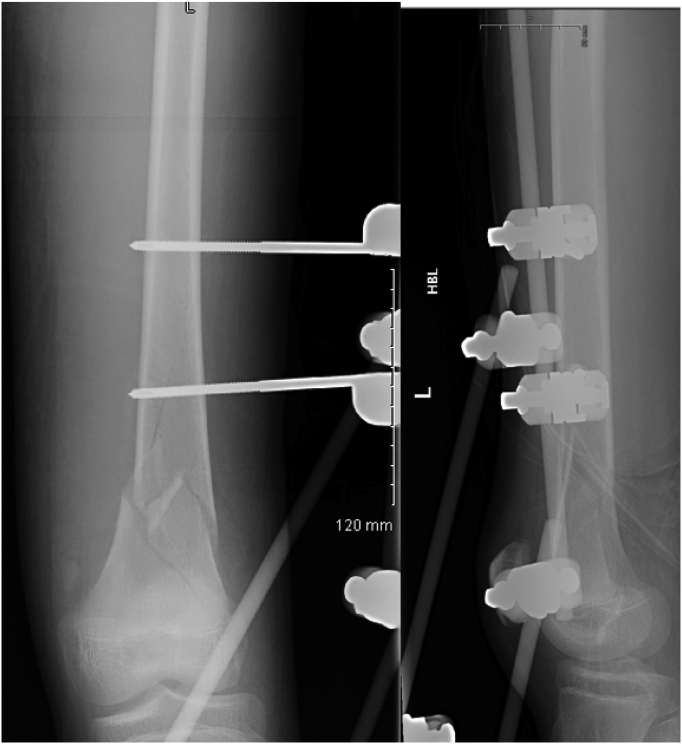
post-operative radiograph of the left distal femur with external fixator in situe.

**Fig. 8:**
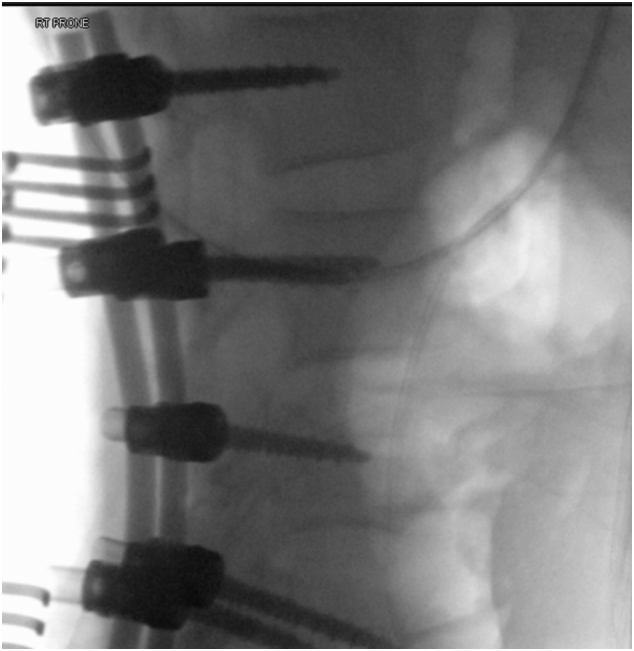
Intraoperative radiograph shows posterior stabilisation from L1 to L5.

The patient was in the supine position on the orthopaedic table. An external fixator was applied to the pelvis and used as a lever arm for closed reduction of the LSP. This involved applying an anterior and lateral pulling force directly on the pubic bone via the external fixator with a simultaneous external rotation force applied on the left iliac wing (Fig. 5). The bilateral SIJ fracture was fixed with two fully threaded cannulated screws (Fig. 6) using the method described by Routt et al. [21]. The distal femur was fixed with external fixators (Fig. 7). The patient's position was then changed to prone for posterior spinal stabilisation L1 to L5 with two rods and ten pedicular screws (Fig. 8).

**Fig. 9:**
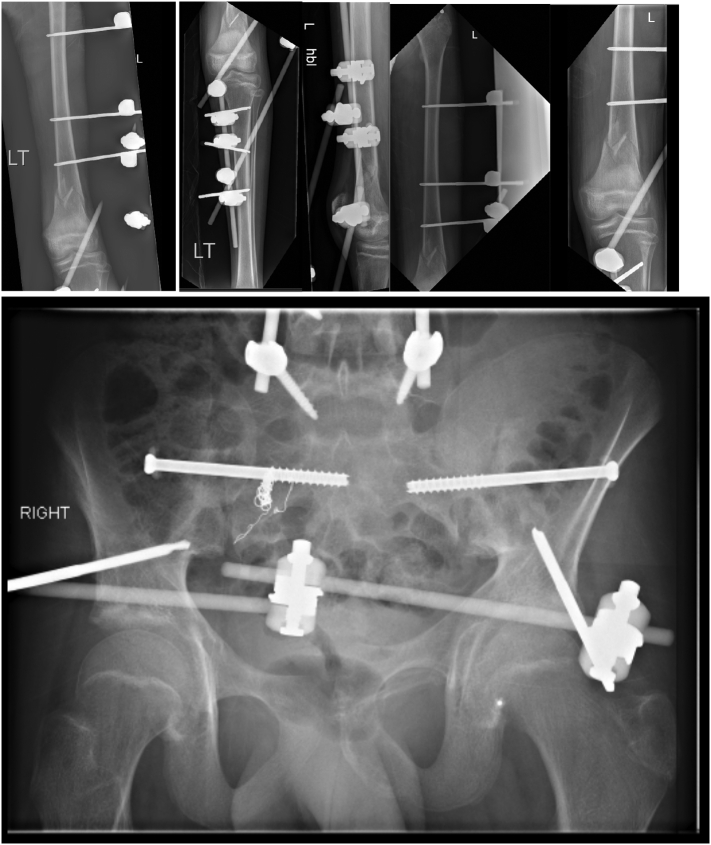
Post-operative radiograph (eight weeks) of the pelvis and left femur, shows posterior stabilisation from L1 to L5, external fixation in situ of the pelvis and left femur.

**Fig. 10:**
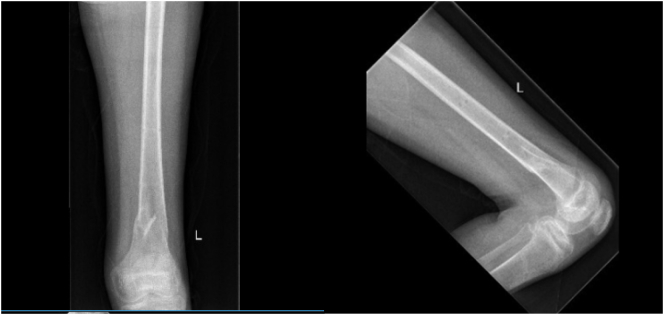
post-operative radiograph of the left distal femur after removal of external fixator (8 weeks).

Post-operative management included analgesia, thromboembolic prevention commenced 48 h post operatively with subcutaneous enoxaparin, bed rest for 48 h with 30 degrees inclination. The post-operative period was uneventful. We allowed partial weight bearing for eight weeks. We removed the external fixator after eight weeks from the initial surgery after clinical and radiological confirmation of fractures healing by plain radiograph (Fig. 9, Fig. 10).

### Follow up after one year

**Fig. 11:**
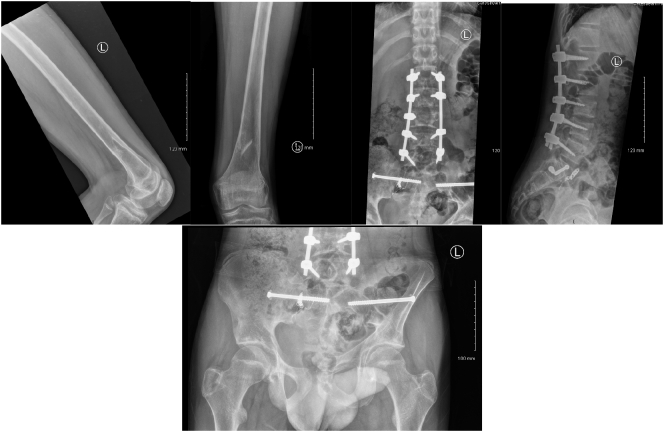
post-operative radiograph (after one year) of the lumbar spine, pelvis and left femur shows satisfactory healing and alignment.

After one year from the initial injury the patient was fully weight bearing with no limping. We assessed his quality of life and mobility by SF-12 an SF-32 outcome scores [22]. His SF-12 was 42 and SF-32 was 97. His updated radiographs are illustrated in (Fig. 11).

## Discussion

From the available literature, LPS is an exceedingly rare forms of pelvic injury. LPS is also referred to as overlapping pubic symphysis. These injuries are more common in males with male to female ratio approximate at 11:3 [9]. Some authors described the female pubic symphysis with a thicker cartilaginous disc and 2–3 mm greater mobility than the male pelvis. This contributes to its stability contrasted to the male pelvis [6]. The LSP can either be anterior or posterior [1]. As in our case, most reported cases in the literature were locked posteriorly [1,5,10,17,23]. Of note, both skeletally mature and immature children who sustain pelvic fractures have injuries to multiple body organ systems with variable seriousness. Shaath et al., suggested that patients with an open triradiate cartilage are unique. Patients with a closed triradiate cartilage should be managed as adults. Irrespective of skeletal maturity, it is crucial for emergency, critical care teams and orthopaedic surgeons to classify pelvic fractures in paediatric patients as markers of high energy trauma, with a necessity to pursue the associated injuries [24]. Current trauma literature recommends to avoid pelvic packing for this type of internal bleeding control in paediatrics [21]. It can lead to further bleeding as opening the peritoneum will release the tamponade. Moreover, the funnel shape pelvis in paediatrics does not allow for packing to remain in place to provide a compressive haemostatic effect [25,26]. Most reported cases of LPS were hemodynamically stable in keeping with the mechanism of lateral compression [9]. However, in our case, their was internal pelvic bleeding,as our patient was haemodynamically unstable.

Simple LSP can usually be reduced closed. Some authors described the way closed reduction by using the femur as a lever arm in flexion, abduction and external rotation to tighten the iliofemoral ligament. Then, gentle rocking motion in the form of rotation and abduction of the femur may reduce the overlapped dislocated pubic symphysis [4,5,10]. however, it has a risk of iatrogenic fracture of the ipsilateral femur. Moreover, it is hard to achieve closed reduction if the LSP was associated with ipsilateral femoral fracture as in our case [2]. Hence, we used the external fixator not the femur as a lever arm for closed reduction.

Most authors advocate for fixation of the sacrum by the percutaneous method using ISJ screw [6,9,21]. The reduction was stabilised using a trans-symphyseal screw of 60 mm × 4.5 mm, after external fixation with Hoffman external fixator. Treatment of trans-symphyseal instability using an internal plate fixation is the most common procedure for adult patients described in the literature [27]. However, other techniques such as application of hip Spica for four weeks after open reduction have been described [28]. In a case of failed reduction, a superior ramus osteotomy was performed on the contralateral hip to aid reduction [10]. LSP in the paediatric patient should be treated by closed reduction to unlock the LSP and reduce the pelvis with an external fixator simultaneously with fixation of the ISJ by two illiosacral cannulated screws.

## Conclusion

Locked symphysis pubis in the paediatric patient is an exceedingly rare injury among lateral compression type pelvic fractures. Careful assessment and a preoperative management plan are encouraged. Open packing of the pelvis in case of internal bleeding should be avoided in this patient cohort, advocating for the use of selective embolization instead. Closed reduction of the LSP can be successful using the external fixator as a lever arm for reduction followed by percutaneous fixation of the SI joint.

For posterior lumbar spine stabilisation patient position can be changed to prone intra-operatively to complete management during the index surgery.

### Learning points

• Locked symphysis pubis in the paediatric patient is an exceedingly rare injury among lateral compression type pelvic fractures.
• Closed reduction of the LSP and percutaneous fixation of the SI joint followed by posterior lumbar spine stabilisation is our preferred method of treatment.
• Open packing of the pelvis in case of internal bleeding should be avoided in paediatric patients, selective embolization is advocated.
• A good outcome can be obtained after careful assessment and preoperative management plan.

## References

[1] Sreesobh K.V., Sageer A.M., Raffic M. (2006). Locked overlapping dislocation of the pubic symphysis into the obturator foramen: a case report. J. Orthop. Surg. (Hong Kong).

[2] Afshar A., Koushkzari M. (2015). Overlapped pubic symphysis; a case report and review of the literature. Arch. Bone Jt. Surg..

[3] Zwingmann J., Eberbach H., Strohm P.C. (2019). Decision-making, therapy, and outcome in lateral compression fractures of the pelvis – analysis of a single center treatment. BMC Musculoskelet. Disord..

[4] E G.W. (1952). Dislocations of the os coxae. Am. J. Surg..

[5] Ansari S., Rollins J., Ebraheim N.A. (2003). Locked pubic symphysis with ipsilateral fracture neck of a femur. J. Trauma.

[6] Cannada L.K., Reinert C.M. (2009). Case report: locked pubic symphysis: an open reduction technique. Clin. Orthop. Relat. Res..

[7] Thulasiraman V., T.R.R. Pandian SA (2010). Locked pubic symphysis—a case series. Inj Extra.

[8] Yang Q., Wang T., Ai L. (2020). Clinical outcomes of blood transfusion to patients with pelvic fracture in the initial 6 h from injury. Exp. Ther. Med..

[9] Maqungo S., Sa F., Roche S., Sa F. (2010). Case Report and Overlapping Pubic Symphysis Dislocation: A Case Report and Proposal of Reprint Requests.

[10] O’Toole R.V., Sagebien C., Andersen R.C., Nascone J.W. (2006). Superior pubic ramus osteotomy to treat locked pubic symphysis: a case report. J. Bone Jt. Surg. - Ser. A.

[11] Lawal K.O., Clayson A.D., Charalambous C.P. (2013). Open Book Injury Secondary to Doing the Splits. Case Reports 2013:bcr-2012-007870-bcr-2012-007870.

[12] Tile M., Helfet D.L.K.J. (2003). Fractures of the Pelvis and Acetabulum.

[13] Robinson D., Hendel D., Halperin N. (1989). An overlapping pubic dislocation treated by closed reduction. J. Trauma Inj. Infect. Crit. Care.

[14] Webb P. (1977). Overlapping dislocation of the symphysis pubis: a case report. J. Bone Jt. Surg. Am..

[15] Shanmugasundaram T.K. (1970). Unusual dislocation of symphysis pubis with locking. A case report. JBJS Am..

[16] Wilson J.N. (1982). Watson-Jones Fractures and Joint Injuries.

[17] Gordon R.O., Mears D.C. (1991). Lateral compression injury of the pelvis. A case report. J. Bone Jt. Surg. Am..

[18] Agha Riaz A., Borrelli Mimi R., Farwana Reem, Koshy Kiron, Fowler Alexander J., Orgill SG Dennis P. (2018). The SCARE 2018 statement: updating consensus Surgical CAse REport (SCARE) guidelines. Int. J. Surg. Actions.

[19] Surgeons AC of (2015). ATLS - Advanced Trauma Life Support.

[20] British Orthopaedic Association (2018). The Management of Patients With Pelvic Fractures.

[21] Chip Routt M.L., Kregor P.J., Simonian P.T. (1995). Early results of percutaneous iliosacral screws placed with the patient in the supine position. J. Orthop. Trauma.

[22] Lin Y., Yu Y., Zeng J. (2020). Comparing the reliability and validity of the SF-36 and SF-12 in measuring quality of life among adolescents in China: a large sample cross-sectional study. Health Qual. Life Outcomes.

[23] Tadros A.M., PO K. Lunsjo (2009). Overlapping dislocation of the pubic symphysis: report of three cases and review of the literature. Arch. Orthop. Trauma Surg..

[24] Shaath M.K., Koury K.L., Gibson P.D. (2017). Analysis of pelvic fracture pattern and overall orthopaedic injury burden in children sustaining pelvic fractures based on skeletal maturity. J. Child. Orthop..

[25] Fleming S., Berwin J., Patel B. (2015). Pelvic trauma. Abdom. Inj. Risk Factors, Manag. Progn. 04:57–16.

[26] Velmahos G.C., Mattox K.L., Moore E.E.F.D. (2013). Pelvis.

[27] Willy C., Schmidt R., BF H. Gerngross (2004). Treatment of trans-symphyseal instability report., with an internal fixator. Outcome of a surgical technique on the basis of a case. Unfallchirug J..

[28] Shanmugasundaram T.K., Shanmugasundaram T.K. (1970). Unusual dislocation of symphysis pubis with locking. A case report. J. Bone Joint Surg. Am..

